# Legislation for Midwives.—A Reply

**Published:** 1900-06-16

**Authors:** 


### Editor's Letter-Box.

[Our Correspondents are reminded that prolixity is a great bar to publication, and that brevity of style and conciseness of statement greatly facilitate early insertion.]

### LEGISLATION FOR MIDWIVES.—A REPLY.

Sir,—Perhaps, as you have opened your columns to a discussion on the midwifery crux, you will permit me a rejoinder to the correspondent who has lately been handling it. I observe that two features of the present Bill are the main objects of his animadversion, for we may pretermit the consideration of certain very small stumbling blocks which require very keen sight to discern at all. We are told first of all that the Central Board of Midwives will be a failure and a sham, because the lying-in hospitals, or midwifery schools, as your correspondent prefers to call them, have no representation on it. But where do we find it stated that they will have no representation? Far be it from me to depreciate for one moment the ability of any writer in your columns. I admit his discernment, and his care for the generation yet unborn has filled me with admiration. Would that the election of this Board lay altogether in his hands, then all fears might be allayed and the nation could lay down in peace. But since this cannot be done, let me at least lean on a staff which has borne me before, and has taken some years to mature. Let me be pardoned if I admit a confidence in the common-sense of the bodies, on the whole respectable and well meaning, to whom this business of selecting the delegates has been assigned. And to this I incline the more inasmuch as writers—even writers in The Hospital—though admittedly omniscient, are not yet endowed with immortality, whereas to all present seeming her Majesty's Privy Council and the Royal Colleges of Surgeons and Physicians are likely to endure so long as midwives shall be needed in the land. Moreover, would it not puzzle all the wise men of Gotham together to settle how your "Midwifery School" are going to agree upon a delegate? When did they ever cohere? What joint action have they ever compassed? Does your correspondent suppose that they are even aware of the existence one of another? Pray Heaven we be not to wait for a Bill till your lying-in hospitals have leisure to form a league of brotherhood. It were surely a lesser evil to leave the Bill as it stands and trust that the gentlemen responsible for the Board will equal the wonderful sagacity of your correspondent in appreciating the fact that those who are appointed to serve on it might with advantage be men possessing some small knowledge of their business. But there is a second grievous error in the framing of the Bill it seems. We are threatened with a reign of darkness in matters maternal because our midwives for the future are to have but three months' training or are to be allowed to flourish the L.O.S. certificate without any training at all. But, sir, believe me there is no word of this or of any standard of qualification at all in the Bill now before Parliament. The three months' midwife is a figment of your correspondent's brain. It might lay a somewhat embarrassing burden on the right hon. gentlemen if they were to be called on to settle a scheme of education for ladies of this ancient calling. And some impatience might be evinced by our legislators were they required from time to time to modify the curriculum in accordance with later theories. No, sir, all that Parliament can be asked to do in this direction is to safeguard those who are already earning their bread as midwives and to sanction the formation of a board charged with regulating their education for the future. Your correspondent would have Parliament sanction the creation of a superior order of midwives. Nothing I maintain could be more mischievous. It does not appear that he intends his higher class midwives to know more of their business than their humbler sisters; they are merely to boast a longer acquaintance with the details of hospital work. Yet to confer additional privileges would certainly be to convey an impression of additional qualifications. Your privileged midwife would be more than human if she did not feel herself uplifted somewhat in the exercise of her craft, and yet it is not proposed that her attainments shall be a whit less shallow than that of her fellow labourers. We have here a distinction without a difference, and a very dangerous distinction it might prove to be. If these ladies desire to be taken seriously as proficient in their chosen line of business let them do as the medical practitioner does; let them study to some purpose, and not grudge five or six years for the mastery of what might then with some reason be styled their "profession." Your correspondent may not be aware that some such vision of a higher order of midwifery once visited the wise brain of no less honoured an authority than Miss Florence Nightingale. There is extant a little pamphlet in which Miss Nightingale develops a scheme for the education of women who should undergo a course of studies in obstetrics exactly similar to the course compulsory on medical practitioners. These were days before women doctors were in being. They were to begin and end their curriculum as specialists in this one branch of practice, but within these limits their training was to be severe and complete. An obstetrician without general knowledge of medicine or surgery is at the present moment an inconceivable anomaly. But surely an anomaly even more monstrous would be a higher order of midwives, endowed with special privileges, yet possessing (to quote your correspondent's words) no higher qualification than the certificate of the Obstetrical Society of London, or that of the Royal College of Physicians in Dublin. For no one can suppose that three years' residence in a general hospital is likely to impart a very deep knowledge of obstetrics. There is now no bar, as in the days when Miss Nightingale wrote, to the acquirement by women of the knowledge necessary to place them on an equality with men in the delivery of women in labour. Let those wh aspire to distinction in this direction set themselves t master their business, and they may chance to find it a more intricate one than the questions set in the L.O.S. examination might lead them to suppose. Failing this straight if rather steep road to knowledge, they had better do their work under very definite regulations, and aspire as little as is seemly when their very moderate attainments are taken into consideration.

